# Concordance of cancer-associated cytokines and mitochondrial DNA deletions in individuals with hepatocellular carcinoma and people living with HIV in Ghana

**DOI:** 10.1186/s12876-025-04399-5

**Published:** 2025-11-11

**Authors:** James Odame Aboagye, Ruth Ayanful-Torgby, Lei Zhou, Prince Peter Wormenor, Vincent Ganu, Kenneth Tachi, Bernard Nii Akrashie Attoh, Miriam Mensah, Timothy Kuuguu, Sedzro Kojo Mensah, George Boateng Kyei, Elijah Paintsil

**Affiliations:** 1 Department of Pediatrics, Yale University School of Medicine, New Haven, CT 06520 USA; 2 Department of Virology, Noguchi Memorial Institute for Medical Research, University of Ghana, Legon, Accra, Ghana; 3 Medical and Scientific Research Centre, University of Ghana Medical Centre, Legon, Accra, Ghana; 4 Department of Immunology, Noguchi Memorial Institute for Medical Research, University of Ghana, Legon, Accra, Ghana; 5 Department of Medicine, Korle-Bu Teaching Hospital, Accra, Ghana; 6 Department of Medicine and Therapeutics, College of Health Sciences, University of Ghana Medical School, University of Ghana, Accra, Ghana; 7 Department of Epidemiology, Noguchi Memorial Institute for Medical Research, University of Ghana, Legon, Accra, Ghana; 8 Departments of Medicine and Molecular Microbiology, Washington University School of Medicine in St. Louis, St. Louis, MO 63110 USA; 9 Department of Pediatrics, Boston University Chobanian & Avedisian School of Medicine, Boston, MA 02119 USA

**Keywords:** Hepatocellular carcinoma (HCC), Human immunodeficiency virus (HIV), Hepatitis, Inflammatory cytokines, Mitochondrial DNA deletion

## Abstract

**Background:**

Hepatocellular carcinoma (HCC) is becoming increasingly prevalent as a non-AIDS-defining cancer closely tied to chronic HIV infection. It is associated with increased secretion of inflammatory cytokines, immune system dysfunction, and alterations in mitochondrial function. The objective of this study was to investigate the levels of cytokine secretion and mitochondrial DNA (mtDNA) deletion in people living with HIV (PLWH) compared with individuals diagnosed with HCC without HIV.

**Methods:**

A cross-sectional study was conducted with PLWH and HCC patients recruited from the Korle-Bu Teaching Hospital, Accra, Ghana. Participants donated whole blood for the isolation of plasma and peripheral blood mononuclear cells (PBMCs) for analysis. Cytokines were quantified in plasma samples using ELISA and Luminex techniques, while mtDNA deletions were determined with DNA extracted from the PBMCs.

**Results:**

The study found that the secretion of the cytokines TGF-β, FGF2, IL-8, TNF-α, VEGFA, and RANTES was similar in patients with HCC and PLWH. These cytokines have been implicated in HCC initiation and are also elevated in the early stages of the disease. Moreover, we observed comparably high levels of mtDNA deletion in PLWH and HCC patients.

**Conclusions:**

These findings underscore the risks associated with HCC development in PLWH. There is a need for screening among PLWH, and these differentially expressed cytokines could serve as potential biomarkers.

**Supplementary Information:**

The online version contains supplementary material available at 10.1186/s12876-025-04399-5.

## Introduction

People living with HIV (PLWH) are at high risk of developing acquired immunodeficiency syndrome (AIDS)-defining cancers such as Kaposi’s sarcoma, non-Hodgkin lymphoma, and cervical cancer [1]. The advent of antiretroviral therapy (ART) has reduced the incidence of AIDS-defining cancers, leading to a reduction in morbidity and mortality and an extended life expectancy [2, 3]. Despite the benefits of ART, the incidence of non-AIDS-defining cancers in PLWH, such as hepatocellular carcinoma (HCC), Hodgkin’s lymphoma, skin melanoma, and oral, lung, and anal cancers, is increasing [2]. A study conducted in the United States of America estimated that the mortality rate for non-AIDS-defining cancers between 2001 and 2015 was 9.2% compared to 5% for AIDS-defining cancers [4]. Further supporting this trend, data from a multicenter study in Europe involving PLWH showed a 15% mortality rate associated with non-AIDS-defining cancers [5].

Hepatocellular carcinoma (HCC) also known as liver cancer is the seventh most common cancer and the second leading cause of cancer deaths globally [6, 7]. In 2020, over 900,000 new cases were reported worldwide, with the highest burden observed in Asia and Africa [8, 9]. Notably, the incidence of HCC is rising among PLWH with an estimated 72% increase in the incidence rate, particularly among those aged 50 years or older [10]. A recent retrospective study conducted in the USA and Canada estimated that the prevalence of HCC in PLWH was between 0.1% and 0.5% [10, 11]. In contrast, studies in HIV-endemic regions in South Africa, Uganda, and Kenya reported a higher prevalence of 18 to 22%, highlighting the enormous burden of HCC among PLWH in sub-Saharan Africa [12–14].

PLWH with chronic hepatitis B or C virus (HBV or HCV respectively) coinfection are at a higher risk of developing HCC [15]. HBV and HCV infections are more predominant in PLWH [16]. Moreover, compared to individuals with HBV and HCV monoinfections, PLWH with HBV and/or HCV coinfections experience rapid progression to end-stage liver disease [11, 17]. These viruses establish chronic infections, triggering persistent production of cytokines by the host immune response [18].

Cytokines, modulated by the host immune system are secreted to regulate cellular stresses, including infection, inflammation, and carcinogen-induced injury [19, 20]. Cytokines are classified as pro- or anti-inflammatory. The imbalance between pro- and anti-inflammatory cytokines in chronic infections established by HIV, HBV, and HCV results in chronic inflammation. The state of chronic inflammation is a critical factor in the formation, development, progression, and metastasis of cancers [19, 21, 22]. Although cytokines are linked to the development of several cancers, certain cytokines are markers for specific cancers. For instance, interleukin (IL) 6 (IL-6), transforming growth factor beta (TGF-β), fibroblast growth factor 2 (FGF2), monocyte chemoattractant protein 1 (MCP-1), and vascular endothelial growth factor A (VEGFA) have been linked to the pre-cancer or initiation (formation) of HCC. The developmental or early stage HCC has been associated with VEGFA, growth differentiation factor 15 (GDF-15), regulated upon activation, normal T cell expressed and secreted (RANTES/CCL5), and osteopontin (OPN), while IL-10, macrophage inflammatory protein 3 (MIP-3), and IL-37b are makers of advanced stage of HCC [19, 23]. Other studies have reported that tumor necrosis factor-alpha (TNF-α), a key marker of cirrhosis and HCC development, is correlated with the secretion of IL-6 [23]. In addition, the secretion of TNF-α is implicated in the production of reactive oxygen species (ROS) by the mitochondria, which results in oxidative stress and nucleotide damage leading to mitochondrial DNA (mtDNA) mutagenesis [24]. Furthermore, there is a preponderance of data to suggest that HIV increases the risk of cancer, although the underlying mechanisms are not well understood. Could pro-inflammatory cytokines play a role in the increased incidence of non-AIDS-defining cancers in PLWH?

The purpose of this study was to determine the levels of cytokine secretion and mitochondrial deletion in PLWH and individuals with HCC without HIV infection in Ghana. We hypothesize that the secretion of cytokines by PLWH results in chronic systemic inflammation and immune dysregulation, which are responsible for the increased risk of HCC. Delineating the cytokines differentially secreted between PLWH and individuals with HCC could be crucial in screening for HCC in PLWH and facilitating appropriate care and treatment.

## Materials and methods

### Study design and study population

This was a cross-sectional study of individuals diagnosed with HCC (HCC+) without HIV infection and PLWH (HIV+) receiving care at the Section of Gastroenterology and the Fever’s Unit, respectively, at the Korle-Bu Teaching Hospital (KBTH), Accra, Ghana. The HIV + participants were further grouped into individuals coinfected with HBV and/or HCV (HIV+/Hep+) or those without hepatitis infection (HIV+/Hep-). Study participants were recruited on convenience during their routine clinic visits between 28th June and 27th September 2022. Inclusion criteria for the study were 18 years and above, individuals with HIV and/or HCC due to virus hepatitis (HBV and HCV). Exclusion criteria were individuals below 18 years, presence of other cancers, and HCC participants with non-viral etiologies including fatty or alcohol-associated liver disease since the study focused on HBV and HCV etiologies.

### Data collection and sample collection

All participants were 18 years and above and provided written informed consent prior to enrollment. At enrollment, 20 ml of blood was collected from each participant. A structured questionnaire was designed to collect sociodemographic, medical and clinical data from participants and medical records. At enrolment, information including age, gender, height, weight, educational level, history of smoking, alcohol intake, chemical/radiation exposure, and family history of cancer were collected directly from the participants (Additional file 1, Part A). Additional information including history of infection, years on ART treatment, HIV stage (WHO criteria), and the most recent data for HIV viral load, HIV CD4 count, and liver function test (LFT) were reviewed and recorded from participants medical records (Additional file 1, Part B). The LFT is routinely done for the participants, specifically, the most recent available test results at the time of each patient’s last clinic visit within the study period were recorded and analysed to reflect their current hepatic dysfunction. The HIV/AIDS staging was assessed based on the World Health Organization (WHO) Clinical Staging System for HIV/AIDS ranging from Stage 1 (asymptomatic) to Stage 4 (AIDS): Stage 1 - Asymptomatic or persistent generalized lymphadenopathy; Stage 2 - Mildly Symptomatic; Stage 3 – Moderately symptomatic; and Stage 4 – AIDS or the severely symptomatic stage [25]. For the HCC participants, treatment and cancer staging was not taken due to information from a recent study reported by Tachi et al. from our study site, the Gastroenterology Unit at KBTH. The study demonstrates that over 70% of the cases had advanced HCC hence, majority received predominantly palliative care, mainly analgesia and supportive treatment. Thus approximately 3% of the patients received sorafenib and microwave ablation. The limited use of these interventions was primarily due to late-stage presentation and the high cost of available treatments. Also, several factors impacted the staging of HCC: (i) The lack of structured surveillance among high-risk individuals limits early detection of asymptomatic tumours; (ii) Cost and limited access to diagnostic imaging, such as contrast-enhanced CT or MRI, which are essential for accurate staging; and (iii) Delayed referral pathways from primary and secondary health facilities to our specialist centres lead to further progression of disease before evaluation [26]. The data were entered and managed using Research Electronic Data Capture (REDCap) software [27].

### Blood separation

Plasma and peripheral mononuclear blood cells (PBMCs) were separated from whole blood through density gradient centrifugation using Ficoll^®^ as previously described [28]. The plasma was stored at −20 °C while the PBMCs were cryopreserved in a freezing medium and stored in liquid nitrogen.

### Serological testing of HBV and HCV

All Participants were screened for HBV (Acro Biotech, California, USA) and HCV (Acro Biotech, California, USA) using the manufacturer’s instructions.

### Cytokine profile

Plasma from each participant was assayed for cytokines using ELISA and Luminex assays according to the manufacturer’s instructions [29, 30]. MIP-3, OPN, GDF-15, and TGF-β were measured using ELISA (Sigma, Darmstadt, Germany). A luminex assay (MILLIPLEX MAP, Millipore, Darmstadt, Germany) was used to measure the expression levels of the following cytokines—FGF2, Interferon-gamma (IFN-γ), interleukin (IL)−2, IL-6, IL-8, IL-10, IL-12p70, IL-12p40, IL-18, interferon-gamma induced protein 10 (IP-10/CXCL10), MCP-1, TNF-α, VEGFA, and RANTES/CCL5. The runs were performed in duplicate on at least two occasions.

### Mitochondrial mutations

To assess the mtDNA common deletion (4977 deletion), nested PCR was performed using primers and protocols as previously described [31]. In brief, genomic DNA was extracted from PBMCs using the DNeasy blood and tissue kit (QIAgen, Hilden, Germany). Two pairs of primers were used for the detection of the 4,977-bp deletion: The round 1 primers were 1 F: AACCACAGTTTCATGCCCATC; and 1R: TGTTAGTAAGGGTGGGGAAGC; and the round 2 primers were 2 F: ACCCTATTGCACCCCCTCTAC; and 2R: CTTGTCAGGGAGGTAGCGATG. The amplification conditions used for the nested PCR was pre-denaturation at 94 °C for 5 min; followed by 30 cycles at 94 °C for 10 s, 58 °C for 45 s, and 72 °C for 50 s; and a final extension at 72 °C for 10 min. The PCR amplicons were visualized on 2% agarose gel electrophoresis and the presence of a 358-bp band was indicative of the presence of 4,977-bp mtDNA deletion.

### Data analysis

Statistical analyses were performed using the Statistical Package for the Social Sciences version 25 (SPSS; IBM Corporation, Armonk, NY) and GraphPad Prism 9 (Dotmatics, Boston, USA). Categorical variables were presented as frequencies and percentages, while continuous variables were expressed as medians and interquartile ranges (IQRs) with confidence intervals (CIs). Chi-square tests and/or regression analyses were used in determining risk-associated factors. Statistical significance was considered *p* < 0.05.

## Results

### Demographic and disease characteristics of the study participants

We enrolled a total of 107 participants, 33 had HCC without HIV (HCC+), 60 were PLWH without HCC (HIV+), and 14 were healthy controls (HIV-/HCC-), as shown in Table 1. Females made up 67%, and the mean age of the study participants was 49 ± 13 years. Approximately 52% were aged 50 years and above. Majority of the participants, 41.1% (between 36.7% and 48.5%) had normal BMI. This was followed by those who were overweight (28.0%; between 18.1% and 35.7%), obese (15.9%; between 0% and 23.3%) and underweight (5.6%; between 0 and 9.1%). Interestingly, none of the HCC + participants were obese compared to the 23.3% in the HIV + participants. The highest level of education was junior high school for 56% of the participants. Nearly half of the participants had a history of alcohol usage. The prevalence of hypertension was higher in HIV + participants compared to HCC + participants. All the HCC + participants were hepatitis B or C positive, whereas 16 out of the 60 (26.7%) PLWH were co-infected with either hepatitis B or C virus (HIV+/Hep+). Study participants with HIV only (HIV+/Hep-) were 44 (73.3%) of the 60 PLWH.

**Table 1:** Demographic and exposure characteristics of the study participants

	HIV+ *N* (%) 60 (56.1)	HCC+ *N* (%) 33 (30.8)	HIV-/HCC- *N* (%) 14 (13.1)	Total *N* (%) 107 (100.0)	*P* value
Gender					
Female	43 (71.7)	15 (45.5)	9 (64.3)	67 (62.6)	< 0.01
Male	17 (28.3)	18 (54.5)	5 (35.7)	40 (37.4)	
Age					
< 50	27 (45.0)	16 (48.5)	13 (92.9)	56 (52.3)	0.31
≥ 50	33 (55.0)	17 (51.5)	1 (7.1)	51 (47.7)	
Mean ± SD	51 ± 11	50 ± 15	37 ± 11	49 ± 13	
BMI (Kg/m ^2^)					
Underweight	3 (5.0%)	3 (9.1)	0 (0.0)	6 (5.6)	0.44
Normal	22 (36.7)	16 (48.5)	6 (42.9)	44 (41.1)	0.54
Overweight	19 (31.7)	6 (18.1)	5 (35.7)	30 (28.0)	0.32
Obese	14 (23.3)	0 (0.0)	3 (21.4)	17 (15.9)	0.01
Missing	2 (3.3)	8 (24.2)	0 (0.0)	10 (9.3)	
Educational Level					
≤ Junior High School	45 (75.0)	15 (45.5)	0 (0.0)	60 (56.1)	< 0.01
> Junior High School	15 (25.0)	18 (54.5)	14 (100.0)	47 (43.9)	
History/Exposure					
Family history of cancer	2 (3.3)	5 (15.2)	4 (28.6)	11 (10.3)	0.04
Hypertension	33 (55.0)	13 (39.4)	0 (0.0)	46 (43.0)	0.15
Smoking	1 (1.7)	2 (6.1)	0 (0.0)	3 (2.3)	0.26
Alcohol	30 (50.0)	19 (57.6)	7 (50.0)	56 (52.3)	0.48
Virus Infection					
HCV	6 (10.0)	7 (21.2)	0 (0.0)	13 (12.1)	0.14
HBV	10 (16.7)	31 (93.9)	0 (0.0)	41 (38.3)	< 0.01
Hepatitis B and C coinfection	0 (0.0)	5 (15.2)	0 (0.0)	5 (4.7)	< 0.01

As shown in Tables 2, 31 (54%) of the HIV + participants had been diagnosed with HIV for at least 10 years while 28 (50%) had been on ART for 10 years. Most of the HIV + participants, were categorized as HIV stage 1 (43.6%), had viral loads less than 50 copies/ml (78.2%), and had CD4 cell counts greater than 200 cells (89.4%). Among the HCC + participants, 18 (54.5%) were males while those aged above 50 years were 17 (51.5%) showing that HCC was fairly distributed between the ages 18 and 49 years and those above 50 years (Table 6). Moreover, only three individuals had been diagnosed with HCC between 1 and 3 years, while the rest had been diagnosed with less than a year, underscoring the low mortality rate associated with individuals with HCC in Ghana (Additional File 2).

**Table 2:** HIV-associated characteristics of the HIV + participants

Variable	HIV+	
	*N* (%)	*P*-value
Years Diagnosed		
< 10	26 (43.3)	0.35
≥ 10	31 (51.7)	
*Missing*	3 (5.0)	
Years on ART		
< 10	28 (46.7)	1.00
≥ 10	28 (46.7)	
*Missing*	4 (6.7)	
HIV Stage		
1	24 (40.0)	< 0.01
2	10 (16.7)	
3	13 (21.7)	
4	8 (13.3)	
*Missing*	5 (8.3)	
Viral Load		
≤ 50 Median (Range)	43 (71.7) < 20 (< 20–30)	< 0.01
> 50 Median (Range)	12 (20.0) 5731.5 (52–2.5e5)	
*Missing*	5 (8.3)	
CD4 count		
< 200 Median (Range)	5 (8.3) 159 (21–183)	< 0.01
200–499 Median (Range)	17 (28.3) 383 (202–495)	
> 500 Median (Range)	25 (41.7) 721 (502–1353)	
*Missing*	5 (100.0)	

### Liver function of study participants

Liver function tests including alanine transaminase (ALT), aspartate aminotransferase (AST), alkaline phosphatase (ALP), albumin, and bilirubin were extracted from the participants’ medical records. The records showed that the HCC + participants had higher levels of the liver enzymes ALT, AST, and ALP as well as bilirubin than the HIV + participants. However, the albumin level was lower in the HCC + participants compared to the HIV + participants (Table 3).

**Table 3:** Liver function tests (liver enzymes and proteins) in the HIV + and HCC + cohorts

Variable	Study Cohort		*P* value
	HIV+	HCC+	
ALT (IU/L)			
Median (Range)	22 (7–123)	62 (17–553)	< 0.01
Normal Range	7 to 55		
AST (IU/L)			
Median (Range)	32 (17–106)	91 (19–870)	< 0.01
Normal Range	8 to 48		
ALP (IU/L)			
Median (Range)	99.5 (0–323)	207 (91–1581)	< 0.01
Normal Range	44 to 147		
Albumin (g/L)			
Median (Range)	41 (4.3–267)	35 (21–46)	0.04
Normal Range	34 to 55		
Bilirubin (µmol/L)			
Median (Range)	7.9 (0–73)	23.1 (4–680)	< 0.01
Normal Range	1.7 to 20.5		

### Cytokine profiles of the study participants

**Fig. 1:**
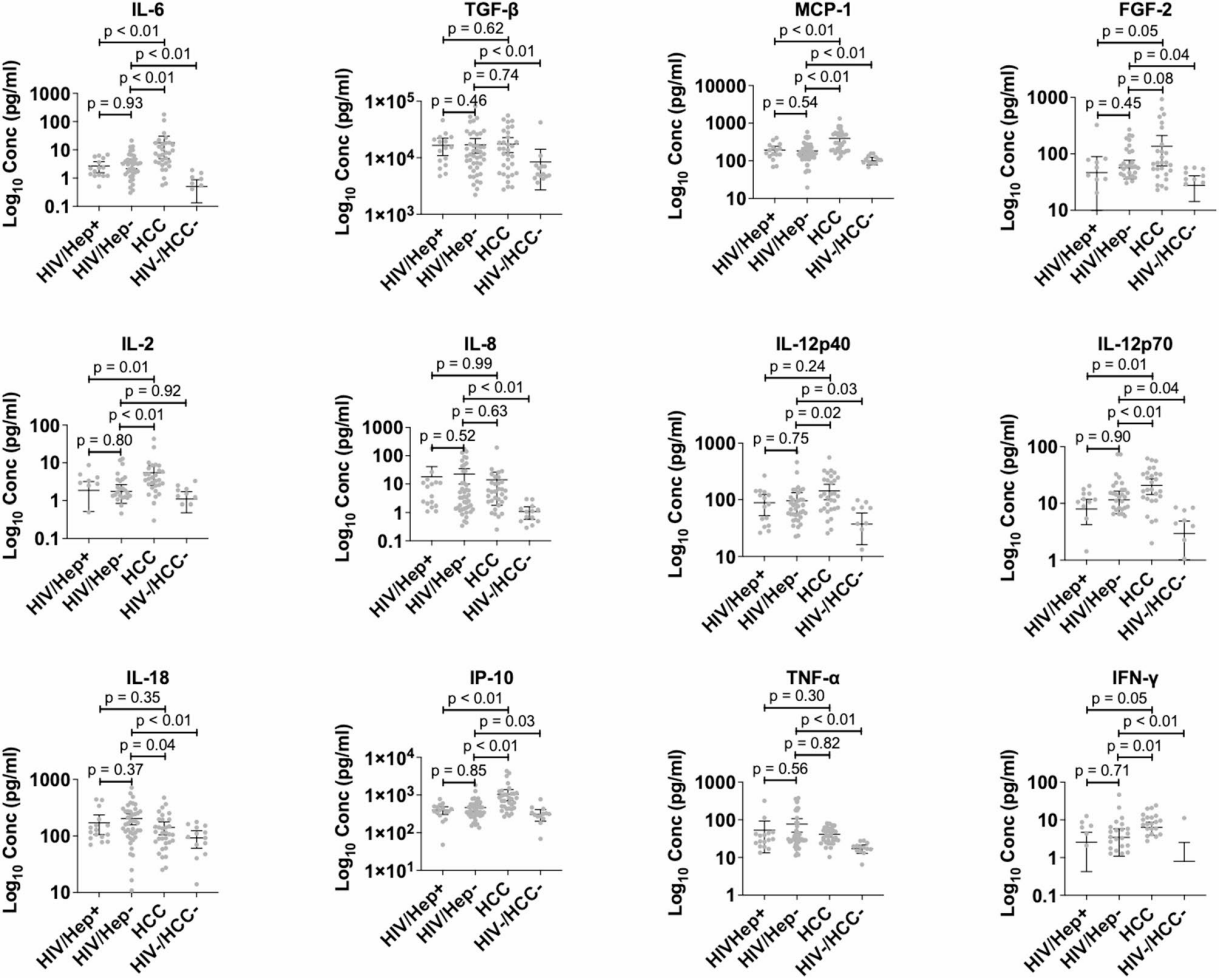
Expression of inflammatory cytokines associated with pre-cancer and initiation of HCC in HCC + compared to HIV + participants coinfected with or without hepatitis B and C viruses. The samples were run in duplicates. The scatter plots with error bars were represented as mean and 95% CI respectively. Differences between groups were tested by the Mann-Whitney U test and significance was considered at p (p-value) < 0.05

**Fig. 2:**
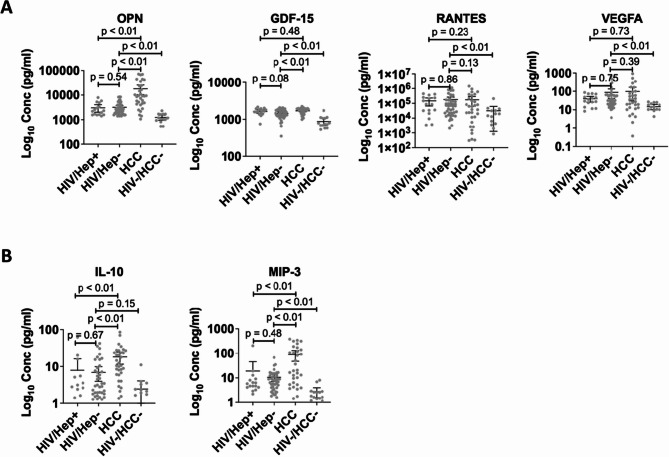
Expression of cytokines implicated in the early and advanced stages of HCC in HCC + compared to HIV + participants coinfected with or without hepatitis B and C viruses. **A** Cytokines implicated in the early or developmental stage of HCC. **B** Cytokines implicated in the advanced stage of HCC. The samples were run in duplicates. The scatter plots with error bars were represented as mean and 95% CI respectively. Differences between groups were tested by the Mann-Whitney U test and significance was considered at p (p-value) < 0.05

We determined the cytokine profile of HCC + participants compared to that of HIV + participants. We screened for 18 cytokines that were either pro-inflammatory or anti-inflammatory. Next, we grouped them into cytokines associated with pre-cancer or the initiation stage of HCC (IL-6, TGF-β, MCP-1, FGF2, IL-2, IL-8, IL-12p40, IL-12p70, IL-18, IP-10, TNF-α, and IFN-γ), the early stage of HCC (OPN, GDF-15, RANTES, and VEGFA), and the advanced stage of HCC (IL-10, and MIP-3). As shown in Figs. 1 and 2, the expression levels of all the cytokines in the HIV + participants were significant compared to the control group, except IL-2 and IL-10. The cytokines associated with the pre-cancer or the initiation stage of HCC, TGF-β, FGF2, IL-8, and TNF-α were expressed at similar levels in the HIV + participants compared to the HCC + participants (Fig. 1). However, IL-6, MCP-1, IP-10, and IL-12p70 had significantly higher expression in HCC + participants than in HIV + participants. A comparison of cytokines implicated in the early stage of HCC showed that OPN was significantly expressed in the HCC + participants but VEGFA, GDF-15, and RANTES were secreted to similar levels compared to that of the HIV + participants (Fig. 2A). More importantly, among the PLWH, the pre-cancer, initiation, and early-stage cytokines were expressed at similar levels between those with and without hepatitis (Figs. 1 and 2A). The advanced-stage cytokines IL-10 and MIP-3 were significantly expressed in the HCC + participants than in the HIV + participants (Fig. 2B). Since obesity is a risk factor for liver disease and HCC, and we had over 20% of the HIV + participants been obese, we analyzed the cytokine secretion profile in the HIV + participants based on their BMI status (Additional files 3 and 4). Among cytokines implicated in the pre-cancer and initiation of HCC, the proinflammatory cytokines MCP-1, IL-2 and IL-12p70 were significantly secreted in obese individuals compared to their non-obese counterparts. IL-6 and IFN-γ were slightly expressed in the obese HIV + participants than the non-obese individuals (Additional file 3). No significant expression was observed for cytokines implicated in the early and advanced HCC stages in either obese or non-obese HIV + individuals.

### Correlation of cytokine levels in HIV + participants

We next investigated the correlations among the 18 cytokines screened. As presented in Table 4, the notable pairwise correlations with the HIV + cohort were 0.89 for IL-12p70 and IL-12p40, 0.87 for RANTES and TGF-β, and 0.85 for TNF-α and TGF-β. There was a high correlation between the cytokines implicated in the pre-cancer, the initiation stage of HCC, and the early stage of HCC. Notably, the cytokines including IL-6, TGF-β, FGF2, VEGFA, IL-8, TNF-α, and RANTES exhibited positive correlations with each other. Interestingly, GDF-15 was negatively correlated with IL-8, TGF-β, VEGFA, OPN, and RANTES in HIV + participants. Table 5 shows the pairwise correlation of cytokines in the HCC + participants. Among the HCC + cohort, there was a positive correlation of 0.31 for IL-10 and MIP-3 indicative of advanced HCC. The secretion of IL-10 positively correlated with IL-18 (0.53) and TNF-α (0.62) while MIP-3 correlated positively with OPN (0.55) among the HCC + individuals, of which showed no correlation among their HIV counterparts.

**Table 4:** Correlations of cytokines in PLWH

		HIV+																	
		IL-6	TGFb	MCP1	FGF2	VEGFA	OPN	GDF-15	RANTES	IL-10	MIP-3	IP-10	IL-2	IL-8	IL-12p70	IL-12p40	IL-18	TNF-α	IFN-γ
HIV+	IL-6	1																	
	TGF-β	**0.52**	1																
	MCP1	**0.27**	−0.06	1															
	FGF2	**0.56**	**0.69**	0.05	1														
	VEGFA	**0.37**	**0.60**	0.06	**0.46**	1													
	OPN	**0.41**	**0.29**	−0.02	0.11	0.23	1												
	GDF-15	−0.13	**−0.33**	0.17	−0.18	**−0.29**	**−0.26**	1											
	RANTES	**0.37**	**0.87**	0.00	**0.55**	**0.66**	**0.30**	**−0.35**	1										
	IL-10	0.22	0.01	0.17	−0.03	0.02	**0.42**	0.16	−0.03	1									
	MIP-3	0.22	0.09	0.00	0.02	0.11	0.09	−0.12	0.02	−0.07	1								
	IP-10	0.08	−0.03	**0.38**	0.01	0.00	0.03	0.13	−0.03	**0.31**	0.02	1							
	IL-2	**0.57**	0.20	**0.43**	**0.42**	**0.29**	0.21	0.00	0.22	0.24	−0.06	0.21	1						
	IL-8	**0.43**	**0.64**	**−0.28**	**0.63**	**0.32**	0.20	**−0.28**	**0.49**	−0.14	0.09	−0.07	−0.12	1					
	IL-12p70	**0.56**	0.24	**0.52**	**0.25**	**0.33**	**0.29**	0.02	**0.26**	**0.47**	−0.10	**0.32**	**0.76**	−0.10	1				
	IL-12p40	**0.59**	**0.26**	**0.52**	0.22	0.25	**0.34**	−0.04	0.23	**0.40**	−0.08	**0.29**	**0.72**	−0.12	**0.89**	1			
	IL-18	0.24	0.22	0.05	0.11	0.07	−0.12	0.06	0.17	−0.01	−0.11	0.13	0.03	**0.35**	0.11	0.02	1		
	TNF-α	**0.52**	**0.85**	−0.08	**0.65**	**0.56**	0.23	−0.19	**0.68**	−0.01	0.06	0.08	0.08	**0.68**	0.12	0.16	**0.27**	1	
	IFN-γ	**0.38**	0.06	0.04	**0.46**	0.22	0.05	0.06	0.07	0.06	−0.04	0.10	**0.73**	0.00	**0.38**	**0.26**	0.12	0.00	1

The values in bold denote significant correlations of cytokine levels among individuals living with HIV

**Table 5:** Correlations of cytokines in HCC + individuals

		HIV+																	
		IL-6	TGFb	MCP1	FGF2	VEGFA	OPN	GDF-15	RANTES	IL-10	MIP-3	IP-10	IL-2	IL-8	IL-12p70	IL-12p40	IL-18	TNF-α	IFN-γ
HIV+	IL-6	1																	
	TGF-β	−0.13	1																
	MCP1	−0.11	0.28	1															
	FGF2	−0.07	0.26	−0.04	1														
	VEGFA	−0.03	**0.38**	−0.03	**0.47**	1													
	OPN	**0.51**	−0.14	0.14	−0.21	−0.10	1												
	GDF-15	−0.08	0.11	0.15	0.13	0.04	−0.29	1											
	RANTES	−0.09	**0.67**	0.08	0.21	**0.51**	−0.19	0.09	1										
	IL-10	0.16	0.27	**0.49**	0.12	0.13	0.23	0.28	0.09	1									
	MIP-3	0.14	**0.33**	0.24	0.03	−0.06	**0.55**	−0.10	−0.05	**0.31**	1								
	IP-10	−0.04	0.20	**0.67**	−0.05	0.02	0.23	−0.13	0.11	**0.50**	0.29	1							
	IL-2	−0.11	0.01	0.20	**0.73**	0.02	−0.16	0.10	−0.04	0.23	−0.11	0.06	1						
	IL-8	−0.04	0.23	0.23	0.26	0.15	0.22	−0.26	0.14	0.06	0.27	0.06	**0.43**	1					
	IL-12p70	−0.12	0.05	0.25	**0.66**	0.05	−0.14	0.14	−0.02	**0.36**	−0.08	0.16	**0.91**	0.16	1				
	IL-12p40	−0.19	0.16	**0.52**	**0.49**	0.09	−0.20	0.22	0.06	**0.53**	−0.16	**0.35**	**0.78**	0.27	**0.85**	w1			
	IL-18	0.25	0.25	**0.52**	0.03	0.16	**0.37**	−0.04	0.06	**0.53**	**0.39**	**0.41**	0.13	0.20	0.18	0.22	1		
	TNF-α	−0.09	**0.37**	**0.57**	**0.41**	0.29	−0.03	0.21	0.21	**0.62**	0.14	**0.47**	**0.49**	0.25	**0.53**	**0.66**	**0.51**	1	
	IFN-γ	−0.09	0.01	0.19	**0.68**	−0.01	−0.12	0.19	0.04	0.29	−0.10	0.10	**0.91**	0.17	**0.95**	**0.80**	0.10	**0.41**	1

The values in bold denote significant correlations of cytokine levels among individuals with HCC

### Mitochondrial mutations among the cohorts

Mitochondrial mutagenesis has been associated with the development of cancers [32]. Mitochondrial deletion mutations are more prevalent than point mutations [33]. We therefore investigated mitochondrial deletion mutations in the PBMCs of HCC + and HIV + participants. We observed a high percentage of mtDNA deletions in both cohorts; 60% and 64% in HIV + and HCC + participants, respectively. Overall, we observed that mtDNA deletion was associated with an increase in age and male gender. There was no association between mtDNA deletion and the history of alcohol usage (Table 6). Odds ratio analysis (Table 7) showed that age above 50 years (OR 3.3; CI 1.1–9.8), males (OR 4.5; CI 1.1–17.8), diagnosed with HIV over 10 years (OR 3.3; CI 1.1–10.0), use of ART over 10 years (OR 2.1; CI 0.7–6.2), and viral loads above 50 copies (OR 2.2; CI 0.5–9.1) were associated with mtDNA deletions in the HIV + cohort. HIV-hepatitis coinfected participants had higher rates of mtDNA mutagenesis (69%) compared to their HIV-monoinfected participants (57%).

**Table 6:** Frequency of MtDNA deletion in the HIV + and HCC + cohorts

Variable	Study Cohort				
	HIV+	HCC+	HIV-/HCC-	Total	*P* value
mtDNA mutation					
Present	36 (60.0)	21 (63.6)	3 (21.4)	60 (56.1)	0.02
Absent	24 (40.0)	12 (36.4)	11 (70.6)	47 (43.9)	
P value	0.03	0.03	< 0.01	0.08	
Age					
18–39	3/7 (42.9)	4/7 (57.1)	1/9 (6.7)	8/23 (34.8)	0.59
40–49	9/20 (45.0)	5/9 (55.6)	1/4 (25.0)	15/33 (45.5)	0.60
≥ 50	24/33 (72.7)	12/17 (70.5)	1/1 (100.0)	37/51 (72.5)	0.87
P value	0.08	0.69	0.12	< 0.01	
Sex					
Female	22/43 (51.2)	9/15 (60.0)	2/9 (22.2)	33/67 (49.3)	0.18
Male	14/17 (82.4)	12/18 (66.7)	1/5 (20.0)	27/40 (67.5)	0.03
P value	0.03	0.69	0.92	0.07	
Alcohol history					
Yes	18/30 (60.0)	10/19 (52.6)	2/10 (20.0)	30/59 (50.8)	0.09
No	18/30 (60.0)	11/14 (78.5)	1/4 (25.0)	30/48 (62.5)	0.13
P value	1.00	0.13	0.84	0.23	

**Table 7:** Association of MtDNA deletions in PLWH

Variable	mtDNA deletion		OR (CI)	*P* value
	Yes (%)	No (%)		
Age				
< 50	12 (44.4)	15 (55.6)	1	
≥ 50	24 (72.7)	9 (27.3)	3.3 (1.1–9.8)	0.03
Sex				
Female	22 (51.2)	21 (48.8)	1	
Male	14 (82.4)	3 (17.6)	4.5 (1.1–17.8)	0.03
Years Diagnosed				
< 10	11 (42.3)	15 (57.7)	1	
≥ 10	22 (71.0)	9 (29.0)	3.3 (1.1–10.0)	0.03
Years on ART				
< 10	14 (50.0)	14 (50.0)	1	
≥ 10	19 (67.9)	9 (32.1)	2.1 (0.7–6.2)	0.18
Viral Load				
≤ 50	25 (58.1)	18 (41.9)	1	
> 50	9 (75.0)	3 (25.0)	2.2 (0.5–9.1)	0.29
CD4 count				
< 200	1 (20.0)	4 (80.0)	0.1 (0.0–1.5)	0.10
200–499	13 (76.5)	4 (23.5)	1.8 (0.5–7.3)	0.39
> 500	16 (64.0)	9 (36.0)	1	
Hepatitis status				
Positive	11 (68.8)	5 (21.2)	1.7 (0.5–5.6)	0.47
Negative	25 (56.8)	19 (43.2)	1	

## Discussion

The chronic inflammatory state of HBV or HCV stimulates the continuous secretion of cytokines that contribute to HCC development. Moreover, HIV infection leads to chronic inflammation with the secretion of several cytokines (proinflammatory or anti-inflammatory) that could initiate the development of cancers [34]. Consequently, with the increased incidence of non-AIDS-defining cancers including hepatocellular carcinoma, we raised the question: Could pro-inflammatory cytokines play a role in the increased incidence of non-AIDS-defining cancers such as HCC in PLWH? We found high levels of inflammatory cytokines TGF-β, FGF2, IL-8, TNF-α, VEGFA, and RANTES, and mtDNA mutagenesis in PLWH. These cytokines are implicated in the pre-cancerous, initiation, and early stages of HCC. Importantly, the study offered preliminary evidence indicating a resemblance between the secretion patterns of these inflammatory cytokines in PLWH and those observed in HCC patients, thereby underscoring the potential risks of HCC development among PLWH, and hence the increasing incidence of HCC among PLWH.

The levels of cytokines implicated in liver fibrosis, cirrhosis, or the initiation of HCC in HIV + participants were similar to those in HCC + participants with the exception of IL-6 and MCP-1. The cytokines TGF-β, FGF2, IL-8, and TNF-α as observed in the HCC patients and PLWH were consistent with studies implicating these cytokines in hepatic injury and the initiation of HCC in PLWH [35–38]. Moreover, studies have further shown that HIV-infected PBMCs stimulate the expression of pro-inflammatory, pro-fibrogenic, angiogenic, and proliferative cytokines including IL-6, VEGFA, and TGF-β in hepatic stellate cells [39]. The secretion of these cytokines exhibited a positive correlation among the HIV participants, which aligns with findings from other studies indicating a correlation between the cytokines TNF-α, IL-6, and IL-8 in PLWH coinfected with hepatitis, as well as in individuals with chronic hepatitis [37, 40]. TGF-β is anti-inflammatory and involved in inhibiting apoptosis, while TNF-α, IL-8, FGF2, and VEGFA are involved in hepatic injury or cirrhosis through cell proliferative and angiogenic activities [19, 41]. High levels of IL-6 and MCP-1 correlate with worse prognosis for cirrhotic and HCC patients, and hence are suggested as biomarkers to track the development of HCC [42–44]. Furthermore, obesity has been implicated as a factor in liver diseases and HCC which correlates with our findings of higher secretion of IL-6, IL-2, and MCP-1 in PLWH who are obese [45–47]. This suggests that monitoring the secretion levels of IL-6 and MCP-1 could be used to assess liver prognosis in HIV + participants considering comorbid conditions such as obesity. Additional studies are necessary to determine if IL-6 together with cytokines such as MCP-1 could be used to monitor the initiation and progression of HCC in PLWH.

The observed expression of VEGFA and RANTES, both of which are linked to the early stages of HCC development, is consistent with previous findings [48, 49]. Studies have suggested that VEGF and RANTES could predict the presence of HCC and that high levels of these cytokines could be important prognostic factors in determining the survival of HCC patients [50, 51]. Notably, VEGFA is closely tied to angiogenesis, while RANTES has been implicated in facilitating metastasis [50, 51]. These findings underscore the potential role of VEGFA and RANTES as critical players in the early progression of HCC, thereby warranting further investigation into their mechanistic contributions and potential clinical implications.

Furthermore, consistent with other studies, our findings showed that MIP-3 and IL-10 secretions are associated with the advanced stage of HCC [52]. Interestingly, we observed lower levels of MIP-3 and IL-10 secretion among PLWH compared to HCC patients. This finding is consistent with other studies showing high levels of MIP-3 and IL-10 in HCC patients compared to their cirrhotic or non-HCC counterparts [52–54]. The secretion and expression of MIP-3 are further associated with the developmental stages or grades of the liver tumors [55]. MIP-3 and IL-10 are associated with chronic infections with anti-inflammatory effects in immune regulation, angiogenesis, and tumorigenesis [54, 56].

Consistent with other studies, our findings revealed a high rate of mtDNA mutations (deletions) in PBMCs from both HIV + and HCC + participants [57–59]. In addition, the results suggest that hepatitis infection contributes to more mtDNA deletions in HIV-hepatitis coinfected participants than in HIV-monoinfected participants, as demonstrated in another study [59]. The mitochondrion is the chief powerhouse of the cell and is designed to meet the energy and metabolic needs of the cell [32]. However, several environmental conditions, including chronic infections as established in our study participants by the HIV and hepatitis viruses, could disrupt the functions of the mitochondria [59, 60]. The most common causes of disruption of mitochondrial functions are mtDNA mutations, deletions, and impaired DNA replication. The accumulation of mtDNA mutagenesis is a hallmark of mitochondrial dysfunction which is implicated in the development of several cancers including HCC [60]. Subsequently, mitochondrial dysfunction results in the generation of mitochondrial reactive oxygen species (ROS). Mitochondrial ROS can result in further damage to the structure and function of the mitochondria, other organelles, cells, tissues, and organs, contributing to carcinogenesis. Furthermore, chronic infections with the HIV and hepatitis B and C viruses, as observed in the PLWH and HCC participants stimulate the secretion of cytokines such as TNF-α. The secretion of TNF-α stimulates the production of ROS, resulting in oxidative stress. This ROS-generated oxidative stress results in nucleotide damage, leading to mtDNA deletions and subsequently affecting the genome integrity, apoptosis, and promoting the outgrowth of cancerous cells [61, 62]. The biology and interactions of HIV and hepatitis B and C viruses on mtDNA mutations and their implications in the development of HCC are not well understood. Therefore, further studies would be important to determine the role of chronic hepatitis infection in the initiation and progression of HCC in HIV-hepatitis coinfected patients compared with HIV-monoinfected patients.

Our study like any cross-sectional study, has several inherent limitations that we duly acknowledge. First, the inclusion of a well-matched healthy control group, considering gender and age, is essential to establish baseline levels of cytokine secretion in PLWH. Also, the impact of other conditions such as obesity with or without hepatitis in PLWH should be considered. This would enhance the validity of comparisons and interpretations regarding cytokine profiles. Second, this research was an exploratory study, resulting in a relatively small sample size. Although informative, the study design would benefit from the establishment of a larger cohort for longitudinal observation to thoroughly examine the dynamic relationship between inflammatory cytokines and HCC development in PLWH. Third, the absence of ultrasound screening to assess liver conditions in HIV-positive participants, including potential factors such as fibrosis and cirrhosis, is a noteworthy limitation. Correlating such factors with cytokine secretion patterns could provide valuable insights into the interplay between inflammation and liver health. Finally, the study did not assess the correlation between cytokine levels and mtDNA mutagenesis with either the peripheral or tissue viral load of HBV or HCV. The tissue viral load was impossible because it is invasive and not routine standard care in Ghana. This study must be validated in a larger, longitudinal, and well-matched controlled study.

## Conclusion

In essence, HIV infection, regardless of hepatitis coinfection status, appears to trigger the release of cytokines associated with fibrogenesis, cirrhosis, liver tissue tumorigenesis, and mtDNA mutagenesis, which are potentially implicated in the initiation or development of HCC among PLWH. The study presents initial evidence that the profile of these inflammatory cytokines released in PLWH resembles those found in HCC patients, implying an elevated risk of HCC development in PLWH. These findings shed light on the underlying risks of HCC in PLWH and underscore the importance of HCC surveillance within this population. Additional research is imperative to comprehensively investigate and elucidate the roles of cytokines and mtDNA mutations in HCC development, as well as their implications for other cancers within the context of HIV infection and concurrent coinfections with opportunistic viruses.

## Supplementary Information

Supplementary Material 1

Supplementary Material 2

Supplementary Material 3

Supplementary Material 4

## Data Availability

The current data presented in this study are available upon reasonable request from the corresponding author.

## Abbreviations

ART: Antiretroviral therapy
AIDS: Acquired Immunodeficiency Syndrome
FGF: 2–Fibroblast growth factor 2
GDF: 15–Growth differentiation factor 15 (GDF–15)
HBV: Hepatitis B virus
HCV: Hepatitis C virus
HCC: Hepatocellular carcinoma
HIV: Human immunodeficiency virus
IL: Interleukin
MCP: 1–Monocyte chemoattractant protein 1
MIP: 3–Macrophage inflammatory protein 3 (MIP–3)
mtDNA: mitochondrial DNA
OPN: Osteopontin
PLWH: People living with HIV
PBMC: Peripheral blood mononuclear cells
RANTES: Regulated upon activation, normal T cell expressed and secreted
TGF: β–Transforming growth factor beta
TNF: α–Tumor necrosis factor–alpha
VEGFA: Vascular endothelial growth factor A
