# Supplementary material for: Concordance of cancer-associated cytokines and mitochondrial DNA deletions in individuals with hepatocellular carcinoma and people living with HIV in Ghana
Source: BMC Gastroenterol. 2025 Nov 11;25:799. doi: 10.1186/s12876-025-04399-5 (PMC12606890; doi:10.1186/s12876-025-04399-5)
Supplement: Supplementary file 1 — Supplementary Material 1 [file 12876_2025_4399_MOESM1_ESM.docx]

**Data Collection Instrument**

**HIV-HCC Study**

1. **Participant’s questionnaire**

**Participant ID number: ……………. Weight (kg): ………………. Height (m): ….……...**

**1. Age**: …….… (yrs)

**2. Sex** □ F □ M

**3. Highest Educational Level** □ No school □ Primary School □ JSS/Middle School

□ Secondary School □ Poly/Nursing/Teacher Training □ University □ Masters

□ PhD/Professional (doctor, lawyer etc.)

**4. HIV Status** □ Yes □ No

**If no, continue from question no. 7**

**5. Other medical condition(s) or exposure(s)**

A. Rheumatoid arthritis □ Yes □ Used to □ No

B. Organ transplantation □ Yes □ Used to □ No

C. Previous cancer treatment □ Yes □ Used to □ No

D. Chemical exposure (petrochemicals, pesticides, etc.) □ Yes □ Used to □ No

E. Radiation exposure (medical, electrical, etc.) □ Yes □ Used to □ No

F. Family history of cancers □ Yes □ Used to □ No

F. Hypercholesterolemia □ Yes □ Used to □ No

G. Hypertension □ Yes □ Used to □ No

H. Smoking □ Yes □ Used to □ No

I. Alcohol □ Yes □ Used to □ No

1. **Data from medical records**

**6. Years diagnosed with HIV? -------------**

**7. Years on ART?** ……….. (yrs)

□ No treatment □ <1 □ 1-2 □ 3-4 □ 5-6 □ >6

**8. Any malignancies (cancers)?** □ Kaposi Sarcoma □ Non-Hodgkin Lymphoma

□ Invasive cervical cancer □ Hodgkin Lymphoma □ Liver □ Anus □ Oral cavity □ Lung

□ Head and neck □ Others:……………………………………………………………………

**9. Year diagnosed with cancer?** ………………………..

**10. Any virus infection(s) confirmed?**

□ Hepatitis B virus (HBV) □ Hepatitis C virus (HCV) □ Human Papillomavirus (HPV)

□ Human Herpes virus-8 □ Epstein-Barr virus (EBV)  □ Others:………………………..

**11. Year diagnosed with virus infection(s)?** ………………………..

**12. Any bacterial infection(s) confirmed?**

□ Syphilis □ Chlamydia □ Gonorrhoea □ Trichomoniasis

□ Others:…………..……………

**13. Year diagnosed with bacterial infection(s)?** ………………………..

**14. Condition status levels**

A. Viral Load: Baseline……………… Current……………… □ N/A

B. CD4 count: Baseline……………… Current……………… □ N/A

C. BP: Baseline……………… Current……………… □ N/A

D. LDL: Baseline……………… Current……………… □ N/A

E. ALT: Baseline……………… Current……………… □ N/A

F. AST: Baseline……………… Current……………… □ N/A

G. ALP: Baseline……………… Current……………… □ N/A

H. Albumin: Baseline……………… Current……………… □ N/A

I. ALT: Baseline……………… Current……………… □ N/A

**15. Medications prescribed**

A. HIV Infection: ……………………………………………………………………..

B. Viral Infection: ……………………………………………………………………..

C. Bacterial Infection: ……………………………………………………………………..

D. Other Conditions: ……………………………………………………………………..
