# Supplementary material for: Concordance of cancer-associated cytokines and mitochondrial DNA deletions in individuals with hepatocellular carcinoma and people living with HIV in Ghana
Source: BMC Gastroenterol. 2025 Nov 11;25:799. doi: 10.1186/s12876-025-04399-5 (PMC12606890; doi:10.1186/s12876-025-04399-5)
Supplement: Supplementary file 2 — Supplementary Material 2 [file 12876_2025_4399_MOESM2_ESM.docx]

Additional file 2: Association of mtDNA deletion among HCC+ participants

| **Variable** | **mtDNA deletion** | | **OR (CI)** | **P value** |
| --- | --- | --- | --- | --- |
|  | **Yes (%)** | **No (%)** |  |  |
| **Age** | | | | |
| < 50 | 9 (56.3) | 7 (43.7) | 1 | 0.0287 |
| >= 50 | 12 (70.6) | 5 (29.4) | 1.9 (0.4 - 7.9) |  |
|  |  |  |  |  |
| **Sex** | | | | |
| Female | 9 (60.0) | 6 (40.0) | 1 | 0.0342 |
| Male | 12 (66.7) | 6 (33.3) | 1.3 (0.3 - 5.5) |  |
|  |  |  |  |  |
| **Years Diagnosed of HCC** | | | | |
| < 1 | 15 (68.2) | 7 (31.8) | 4.2 (0.3 - 55.6) | 0.266 |
| 1-3 | 1 (33.3) | 2 (66.7) | 1 |  |
| *Missing* | 5 (62.5) | 3 (37.5) |  |  |
