# Supplementary material for: Concordance of cancer-associated cytokines and mitochondrial DNA deletions in individuals with hepatocellular carcinoma and people living with HIV in Ghana
Source: BMC Gastroenterol. 2025 Nov 11;25:799. doi: 10.1186/s12876-025-04399-5 (PMC12606890; doi:10.1186/s12876-025-04399-5)
Supplement: Supplementary file 3 — Supplementary Material 3 [file 12876_2025_4399_MOESM3_ESM.docx]

**Additional File 3: Expression of inflammatory cytokines associated with pre-cancer and initiation of HCC in HIV+ participants associated with BMI.** The samples were run in duplicates. The scatter plots with error bars were represented as mean and 95% CI respectively. Differences between groups were tested by the Mann-Whitney U test and significance was considered at p (p-value) < 0.05.
