# Supplementary material for: Concordance of cancer-associated cytokines and mitochondrial DNA deletions in individuals with hepatocellular carcinoma and people living with HIV in Ghana
Source: BMC Gastroenterol. 2025 Nov 11;25:799. doi: 10.1186/s12876-025-04399-5 (PMC12606890; doi:10.1186/s12876-025-04399-5)
Supplement: Supplementary file 4 — Supplementary Material 4 [file 12876_2025_4399_MOESM4_ESM.docx]

**A**

**B**

**Additional File 4: Expression of cytokines implicated in the early and advanced stages of HCC in HIV+ participants associated with BMI.** (A) Cytokines implicated in the early or developmental stage of HCC. (B) Cytokines implicated in the advanced stage of HCC. The samples were run in duplicates. The scatter plots with error bars were represented as mean and 95% CI respectively. Differences between groups were tested by the Mann-Whitney U test and significance was considered at p (p-value) < 0.05.
